# A remote motion analysis of mass casualty incident simulations

**DOI:** 10.1186/s41077-024-00328-w

**Published:** 2024-12-27

**Authors:** Boris Tolg

**Affiliations:** University of Applied Sciences Hamburg, Ulmenliet 20, 21033, Hamburg, Germany

**Keywords:** Mass casualty incident, Simulation, Movement patterns, Movement correlation

## Abstract

**Background:**

Regular training for mass casualty incidents at physical simulation events is vital for emergency services. The preparation and execution of these simulations consume huge amounts of time, personnel, and money. It is therefore important to gather as much information as possible from each simulation while minimizing any influence on the participants, so as to keep the simulation as realistic as possible. In this paper, an analysis of GPS-based remote motion measurements of participants in a mass casualty incident simulation is presented. A combination of different evaluation methods is used to analyze the data. This could reduce the potential bias of the measurement methods.

**Methods:**

Movement patterns of participants of mass casualty incident simulations, measured by GPS loggers, were analyzed. The timeline of the simulation was segmented into event sections, based on movement patterns of participants entering or leaving defined areas. Movement patterns of participants working closely together were correlated to analyze their cooperation. Written logs created by observers on the ground were used to reconstruct the events of the simulation, to provide a comparative reference to validate the motion analysis.

**Results:**

Recorded motion patterns of the participants were found to be qualitatively related to observer logs and triage allocations, allowing a partial reconstruction of the behavior of the participants during the simulation. By analyzing the times the simulation patients left the site of events some possible misjudgments in the triage decisions were indicated.

**Conclusions:**

Analysis of movement patterns from GPS loggers and comparison with observations made on the ground showed that accurate information about the events during the simulation can be automatically delivered. Although the records of observers on the ground are vital to assess details, delegation of the automated analysis of individual and group motion could perhaps allow observers to concentrate on more specific tasks. The partially automated motion analysis methods presented should simplify the process of analyzing mass casualty incident simulations.

## Background

Mass casualty incidents (MCI) are rare events in which the number of injured persons challenges the capacity of the local emergency services [1]. Although these events are rare, their consequences are severe [2]. It is, therefore, necessary to test protocols and procedures in practical enactments, known as “simulations”, to optimize the management of resources, efficiently treat injuries and save lives. Such simulations are expensive, time-consuming, and require many participants [2]. It is important to gain as much quality information as possible from such events.

In this study, various methods were used to retrospectively analyze a simulated MCI exercise. The exercise was conducted at an airport with the participation of the local fire department, the airport fire department, the police, and a group of professional simulated patients from the German Red Cross. The simulated scenario was the evacuation of an aircraft whose tires had burst after braking on landing.

In order not to disrupt regular flight operations, the fire brigades were unable to approach the accident site directly, creating an artificial element in the exercise. In addition, no real hospitals were available, so a simulated hospital was set up on the grounds of the airport fire department. In the event of such a major incident, it is important for the emergency services to bring order as quickly as possible. To do this, the simulation patients must be divided into different priority categories depending on the severity of their injuries. This process is known as “triage”. The patients should be treated and taken to hospital in this order.

It is therefore important for the emergency services to assign the simulated patients into the correct categories and organize the most efficient transport possible to the hospital. Following a subdivision of Cheng et al. we use simulation as a research method to observe and evaluate events [3]. The aim of the study was to gather as much information as possible during the simulation while disturbing the exercise process as little as possible.

Common methods used to understand how events unfold are logs written in real time by observers, and photographic documentation [4]. Observers on the ground are able to quickly assimilate and distill visual and audible information. However, the information is local and subjective and observers can be distracted or overwhelmed by simultaneous events. Observers can also disturb the simulation [5]. Cameras generate less disturbance but can also deliver only local, and limited, information, particularly if they are stationary. A better overview could perhaps be gained by observation from a drone but local details will be missed.

There remains no standardized approach to analyzing MCI simulations. For example, a combination of cameras, video analytic tools, and radio frequency identification (RFID) sensors on participants was used to define the perimeter of the incident site [6]. A similar system using RFID technology was used to track simulated patients (SPs) [7]. Ingrassia et al. used RFID technology to track the movement of SPs in an MCI simulation [5]. The RFID antennae were placed at entrances to record every unique RFID identifier passing through. The DIORAMA system combines RFID, cameras, and smartphone technology to track SPs and emergency medical services (EMS) in real-time [8–12]. With augmented reality (AR) on a smartphone, patients can be tracked by the EMS, even through walls.

In this article, the methods presented in [13] are further developed with the aim of reducing the required number of observers by using non-invasive global positioning system (GPS) loggers to record the positions of participants. In the open air, it can be assumed that the accuracy of consumer-class GPS systems is 5 m around the actual position [14]. Various analyses of movement patterns were developed, and these were compared with observer logs and triage priorities for patients prescribed by key members of the response team.

It can be assumed that various groups of participants will work together closely, according to their role description during an MCI simulation. Since much communication must be without radios, it is assumed that communication can be interpreted from the movement patterns of these participants.

The general hypothesis is that this information could be used to reduce the number of observers in MCI simulation exercises.

This study addresses the question of whether some decisions and behaviors during an MCI simulation can be identified from the GPS-derived movement patterns of participants. This is tested by comparison with logged observations.

## Methods

The study design presented in this article is a single-arm non-interventional observation of an MCI simulation conducted at the end of 2023. The simulation was realized at an airport and involved a local fire department, the airport fire department, and the federal police force. Twenty-three passengers were evacuated from an aircraft with a simulated burst tire, of whom 5 were in triage category red (vital threat needs immediate treatment), 5 in triage category yellow (seriously injured requires urgent treatment), and 13 in triage category green (slightly injured treatment is not urgent), based on the modified Simple Triage and Rapid Treatment (mSTaRT) triage algorithm. The SPs were members of the German Red Cross, who participated in MCI simulations on a regular basis.

The analysis of the MCI simulation was based on three different data sources.

### GPS loggers

Before the simulation exercise began, numbered GPS loggers were distributed to the emergency services and the simulated patients. The participants’ role was related to the unique GPS logger numbers. Examples of possible roles include Simulated patient (SP) with severe injuries (category red), Driver of an ambulance, or Senior emergency physician. The position data of the participants was recorded every 10 s by the GPS loggers. A total of 82 GPS loggers were used of which, 23 loggers were distributed to simulated patients, 17 loggers to the federal police, 10 loggers to the airport fire department, and 32 to the fire department.

### Observer logs

The exercise was observed by 13 students, some of whom had already gained experience as observers during previous exercises. Some were trained paramedics or members of the fire brigade. They were assigned to specific observation areas and were tasked to write a log of their event observations. Before the exercise began the students were given instructions on how to behave as minimally invasive observers and were given written instructions on what they should pay particular attention to in their area.

The written log contains a minimal amount of information to be compatible with logs from observers of fire departments or the police. They included the hour and minute of the observation, a description of the event, and a category such as: “observation”, “milestone”, “problem” or “solution”.

Some students were instructed to focus on specific tasks, such as the arrival time of patients at the simulated hospital or the behavior of specific roles.

### Patient triage categories

During the planning of an MCI simulation, simulated patients are assigned to roles that have been planned to be in a given triage category. SPs should act out their triage category by representing the severity of their medical condition. These were defined in this simulation exercise by the mSTaRT triage algorithm. If SPs are treated by the response team, they may be assigned to other categories. Also, these assigned categories might change due to further reassessment.

To keep track of the categories assigned to all of the SPs the simulated patients themselves were asked to remember their categories as a backup to the data collected by the observers. The triage results were requested from the SPs by the Red Cross supervisors after the exercise.

#### Confounding factors

The accuracy of the GPS loggers is influenced by the environment. In the open air, the accuracy of consumer-class GPS systems is 5 m around the actual position [14]. Depending on the location of a participant this accuracy may vary.

The participants, the actors, and the student observers are individuals, which can be influenced by interactions with other participants, events during the simulation, weather conditions, or individual characteristics.

The assignment of a triage category depends on many different factors and can be due to the performance of the actor, among other things.

### Movement analysis based on GPS data

The results of all presented methods were combined with the aim of reducing bias in the individual methods.

### Data sources

The data for the statistical analysis is provided by the three methods described. In general, the GPS data is used to create diagrams. The effects visible in these diagrams are validated using the logs of the observers and the triage categories specified by the actors.

#### Time to transport

A parameter “Time to Hospital” [13], was further developed. The original parameter represented patients leaving the site of events and arriving at the simulated hospital. This measurement also includes the transportation time to the simulated hospital. However, as the different simulations provide different times for the transportation of patients to the simulated hospital, this parameter was not well suited to compare results between different simulations.

The modified parameter “Time to Transport” was used to measure the time at which patients left the site of events. Thereby excluding the varying transportation times.

To achieve this, the area of events ${A}_{e}$ was defined. The time at which a simulated patient left the area ${A}_{e}$ was recorded and used for the analysis. Also, the times of arrival of the leading emergency physician and the transport control officer at the event site were recorded and used to create a set of time segments, marking important phases in the handling of the MCI simulation.

#### Movement correlation

The GPS locations of participants consist of three coordinates: The latitude (*y*-direction), longitude (*x*-direction), and altitude (height above ground) [15]. For a flat area of events ${A}_{e}$ the height information can be ignored. The Spearman correlation was used to correlate, pairwise, the two combinations of movement in *x*-direction between participant pairs o_1_ and o_2_ and their movements in *y* (this test was used because such data was not normally distributed [13]). Each combination correlates movements along the *x*- and *y*-axes. Bonett and Wright published a table of sample sizes for the Spearman correlation based on parameter estimation [16]. For a $95\text{\%}\left(\alpha =0.05\right)$ confidence interval and an estimated $\widetilde{\theta }=0.8$ for the correlation of roles that should work closely together, and the width of the confidence interval of $\omega =0.2$ the estimated sample size n equals 72. Since the GPS-data is collected every 10 s, a sample size of 72 equals 12 min. For a more intuitive presentation of the results, we used a sample size of 90 to present the results in 15-min intervals.

The average distance between participants o_1_ and o_2_ was also calculated in 15-min time intervals. In combination with the movement correlation, the average distance makes it possible to identify situations in which the movement is correlated, despite such large distances that direct communication would not be possible.

#### Tactical movement

While the location of SPs was used to evaluate where they had gathered or moved to, additional information was needed for members of the fire departments and the medical staff.

To analyze the tactical movement of the medical staff the simulation area was subdivided into squares of 9 m^2^. The mean direction of all actors of one group within each of these areas was calculated over all timeframes (10 s period), allowing vector plots on a map of the region of the event.

#### Area coverage

2D histograms of the locations of SPs were introduced in [13]. The histograms provide an interpretation of where SPs are predominantly located during the MCI simulation. Thereby it can be assessed whether SPs of different categories are mixed or located separately.

## Results

### Observer logs

The observer logs were used to validate the results of the GPS analyses during special events during the simulation exercise. Two hundred thirty-seven observations were logged and categorized by the observers.

### Patient triage categories

The triage results are shown in Table 1. The first column shows the triage category that was originally assigned, during the planning phase of the simulation. The second column shows the triage category that was assigned to the SP by the paramedics after the first triage. The third column shows the triage category that was assigned to the SPs after a second examination by medics, either during the inspection at the treatment area or in the ambulance.

**Table 1:**
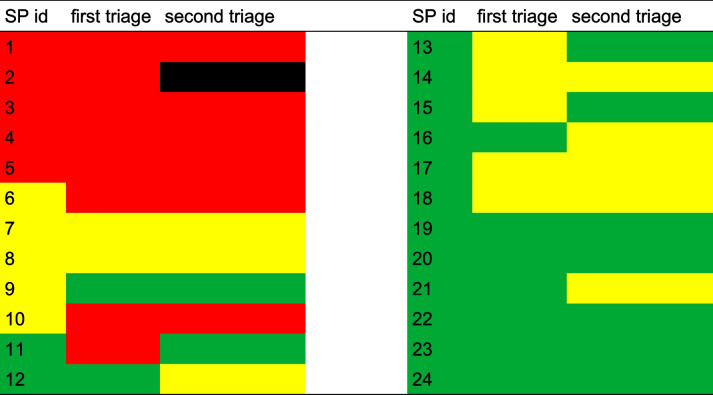
Changes in triage results during the MCI simulation for each SP

The results show that during the first triage, 63% of the SPs were assigned to the pre-planned triage category. Thirty-three percent of the SPs were overtriaged and 4% were undertriaged.

After the second triage, 58% of the SPs were assigned to the intended category, 37% of the SPs were overtriaged, and 4% of the SPs were undertriaged.

### Time to transport

**Fig. 1:**
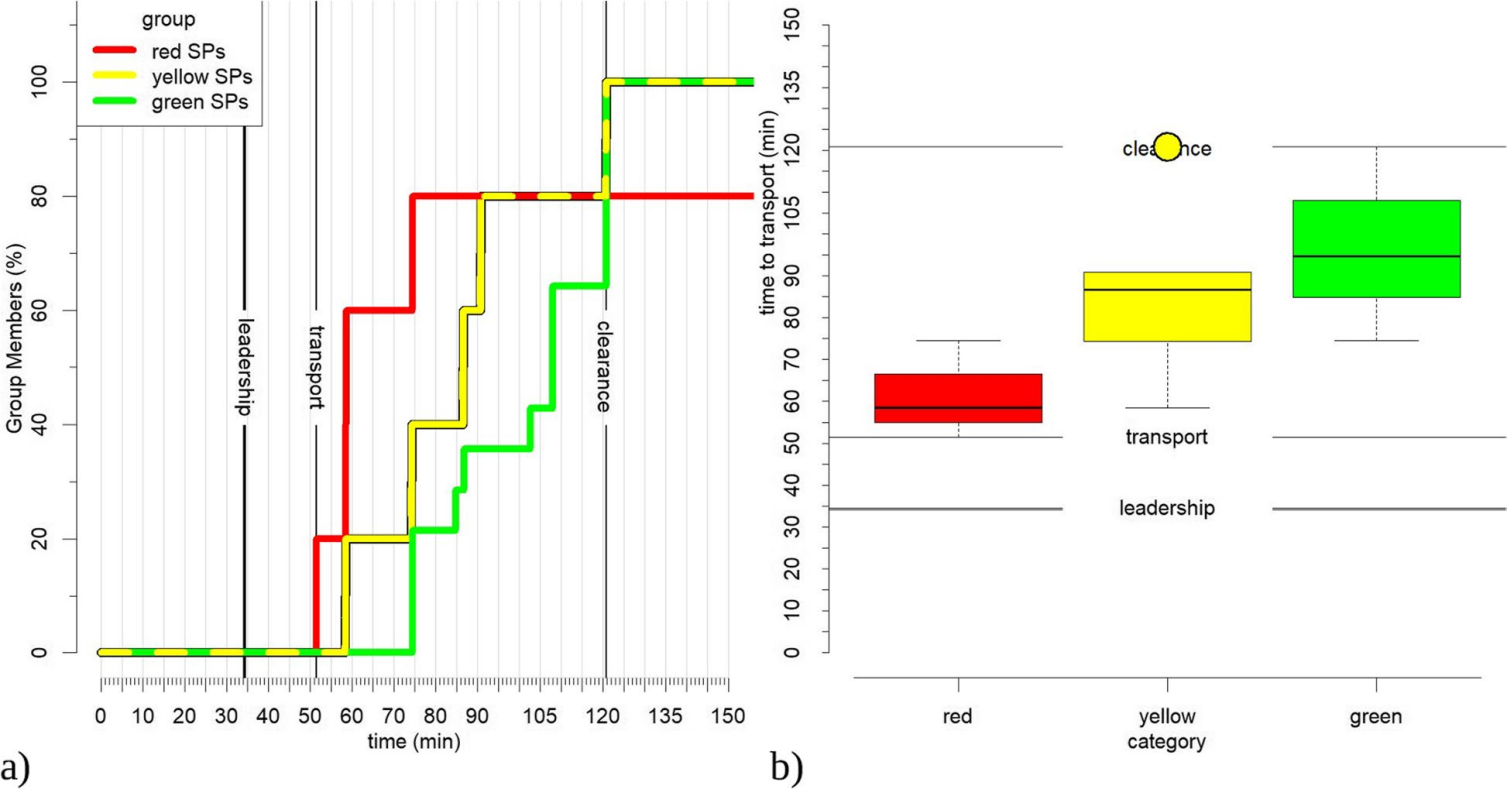
Time to transport SPs to the simulated hospital based on movement data. **a** Red, yellow and green lines show the percentage of SPs of the according category that have left the site of events. The time in minutes is displayed on the *X*-axis. **b** A boxplot of the measured times. Time in minutes is displayed on the *Y*-axis, triage categories are shown on the *X*-axis. Black lines show important time segments of the simulation in both diagrams

The time to transport the SPs to the simulated hospital, based on GPS movement data, is shown in Fig. 1. The triage category of the SPs is based on the intended category assigned during the planning phase. The plot demonstrates that the first patients were transported to the hospital 50 min after the start of the simulation exercise and 17 min after the arrival of senior leadership. All relevant red triage patients (80%) had been taken to the hospital by 75 min, yellow (100%) and green (100%) by 120 min. The 20% failing to reach the hospital in the red group is explained by death. It is observed that there was some overlap in the time of transportation of the triage groups. The first patients of the green group were transported at the same time as the last of the red group and also yellow group patients were transported in parallel with the other groups.

The GPS data shows that the senior emergency physician and the transport control officer (“leadership”) arrived at the site of events 34 min after the start of the simulation. The observer logs at 39 min (Table 2) verifies this GPS information. The logs show that an administrative structure of acting roles had already been created shortly beforehand (31 min to 34 min in Table 2), which had to be revised after the initiation of the full-time management structure with the senior emergency physician and the transport control officer. The boxplot also shows some remarkable information. The circle above the yellow box marks a statistical outlier and is located at the same time that the last SPs of the triage category green left the site of events. This happens around 120 min after the start of the simulation. This might indicate that one yellow SP was categorized as green. The GPS data shows that the outlier was created by SP no. 9. The triage data in Table 1 verifies that SP no. 9 was a yellow SP which was undertriaged.

**Table 2:** Observer logs during the arrival of the senior emergency physician and the transport control officer

Time	Description	Type
31 min	First arriving ambulance hands over the task of triage to acting senior emergency physician and acting transport control officer	Observation
34 min	acting transport control officer talks to fire department (function of person fire department unclear/not marked) about the ambulance holding area/loading zone of the ambulances	Observation
34 min	Acting senior emergency physician comes out of the plane and stands at the foot of the rescue stairs	Observation
34 min	Acting transport control officer organizes the rescue equipment holding area with the airport fire department	Observation
38 min	Acting transport control officer takes off functional vest	Observation
39 min	Arrangements are made between the senior emergency physician and the acting senior emergency physician	Milestone
40 min	The function of the acting transport control officer has been fully transferred to the transport control officer	Milestone
51 min	Organization area for senior emergency physician and transport control officer set up with folding table and chair	Observation
62 min	Transport control officer organizes transport of the injured	Observation
64 min	senior emergency physician and transport control officer discuss with another person from fire department	Observation
66 min	senior emergency physician back in the treatment area	Observation
70 min	senior emergency physician still in the treatment area	Observation
75 min	Organization area for senior emergency physician and transport control officer discuss again	Observation

After 58 min a group of yellow and red SPs left the site of events. The movement data shows that the yellow SP had the ID 10. The triage log verifies that this SP was overtriaged (Table 1). Also, after 75 min a group of green, yellow, and red SPs left the site of events, visible in both charts, which indicates another problem with the triage results. The movement data shows that the three green SPs have the IDs 11, 14, and 15. The triage data verifies that all of them have been overtriaged. The yellow SP had the id 6, which was also overtriaged (verified by Table 1).

The next group of yellow and green SPs can be seen at 87 min. The green SP had the ID 17. The log of the triage categories verifies that this SP was overtrained (Table 1).

Finally, the red SPs did not reach 100% clearance. The observer logs and the movement data verify that the red SP with the ID 2 was declared dead.

### Movement correlation and tactical movement

**Fig. 2:**
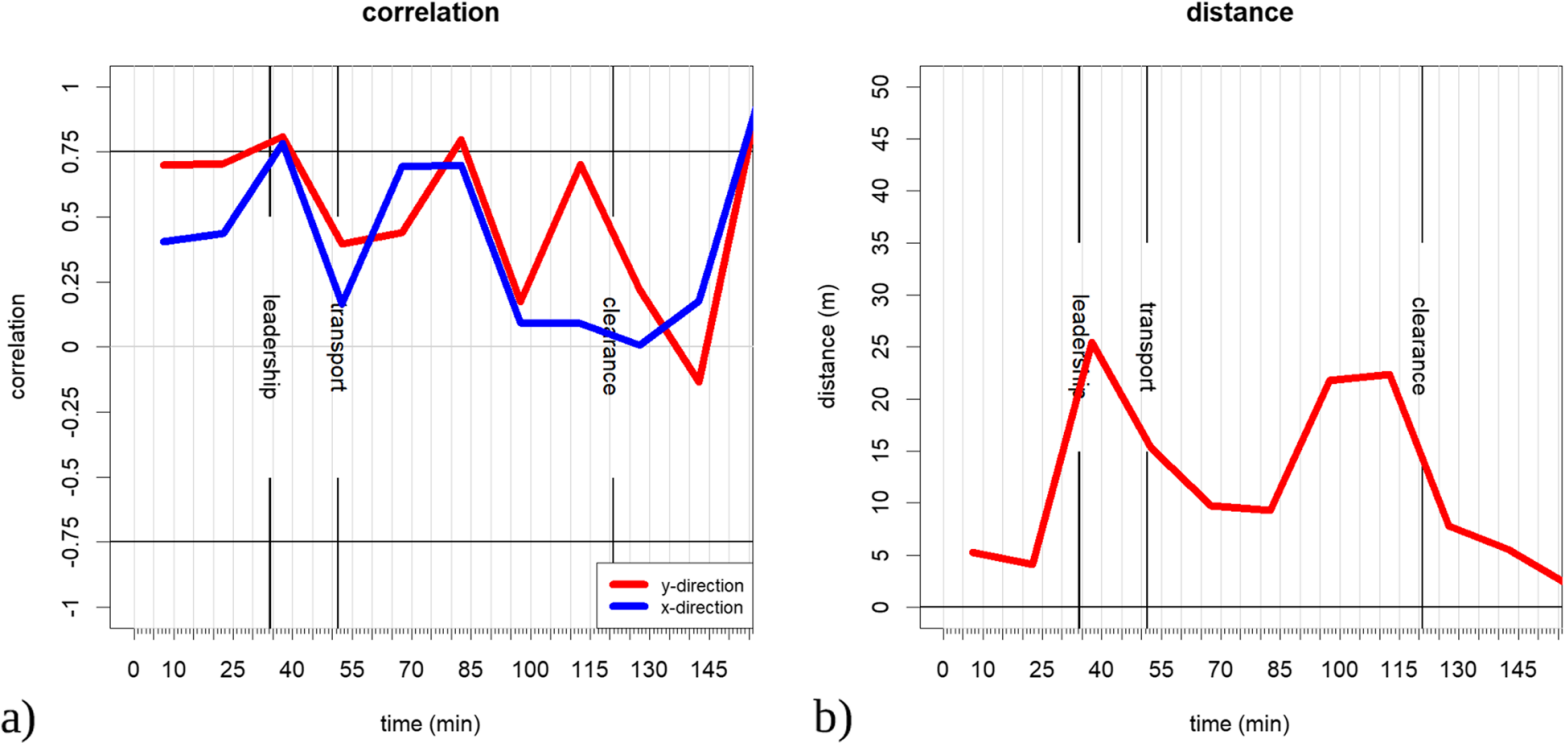
**a** Movement correlation between the senior emergency physician and the transport control officer in *x*- and *y*-direction. **b** the average distance between the senior emergency physician and the transport control officer. Values prior to the leadership segment can be ignored, since they represent artificial behavior due to the simulation planning

The movement correlation and the distances between the senior emergency physician and the transport control officer are shown in Fig. 2a) shows the correlation between the two roles. The *X*-axis of the chart displays the time in minutes, and the *Y*-axis the Spearman correlation values. Values above 0.75 or below − 0.75 are considered a high correlation and indicate an aligned movement. Positive values indicate a simultaneous movement in the same direction, negative values indicate a simultaneous movement in opposite directions. The correlation was calculated in 15-min steps starting from the beginning of the simulation.

Figure 2b shows the average distance between both roles in 15-min time segments. It is observed that the senior emergency physician and the transport control officer are closest at the beginning and end of the simulation exercise when all participants were gathered at one spot. During the exercise, they were closest during the middle of the transport period and most separated at their arrival and slightly before the clearance phase.

Vertical lines in both charts mark the time segments used in Fig. 1.

**Fig. 3:**
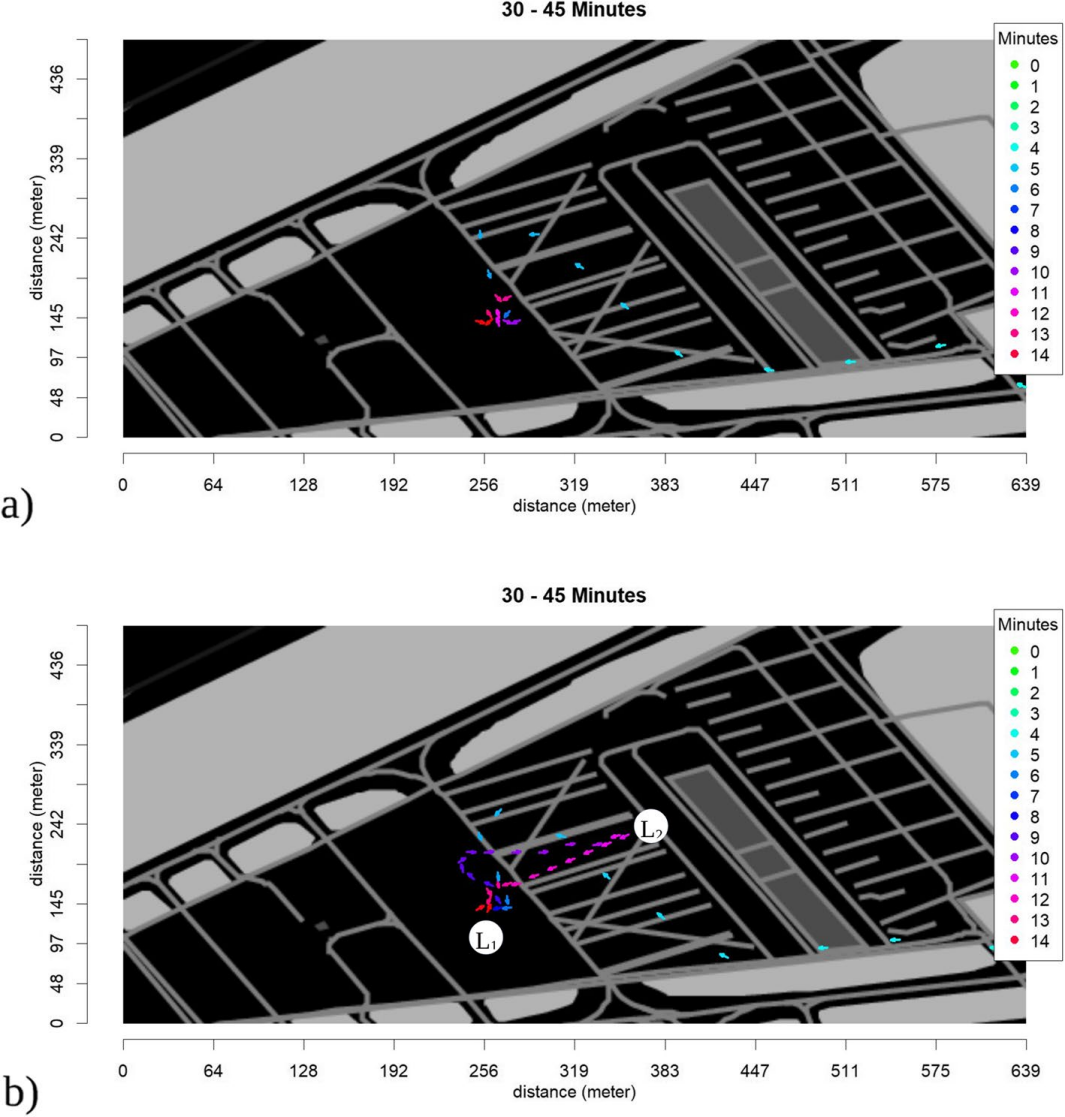
Tactical movement in the time segment from 09:30 am to 09:45 am **a** of the senior emergency physician and **b** of the transport control officer. The *X*- and *Y*-axes show the length of the area in meters. The color codes the time of the movement within the depicted timeframe

The tactical movement shown in Fig. 3 can be used to support the analysis of the movement correlation. The observer logs regarding the senior emergency physician and the transport control officer shown in Table 2 are used for verification. The direction vectors of the senior emergency physician and the transport control officer are shown in Fig. 3a, b, respectively. The period represented begins just before the “leadership” marking at 37 min in Fig. 2, and ends during the middle of the “leadership” period.

Comparing Fig. 3a and b, both roles are observed to arrive on the same route at the site of events (turquoise vectors). While the senior emergency physician (Fig. 3a) stays at one location L_1_, the transport control officer (Fig. 3b) walks in a loop to location L_2_ and back to the senior emergency physician at location L_1_. The color of the vectors in Fig. 3b indicates that the transport control officer arrives at L_2_ approximately 40 min after the start of the simulation which corresponds to the observed handover of his role from his acting counterpart at the same time (Table 2).

A coordinated approach results in a high correlation in the movement patterns. The average distance (Fig. 2b) shows that both roles followed different paths after arrival with increasing distance.

The observer logs show that until 75 min after the start of the simulation, both roles have been working independently with the exception of a joint discussion with a member of the fire department 64 min after the start of the simulation (Table 2).

A comparison with the GPS data shows that both roles are moving around location L_1,_ with similar but not identical movement patterns. The distance between both roles is close. Seventy-five after the start of the simulation both roles were logged as having a joint discussion again. This common behavior is shown as a peak in the movement correlation and a close distance. Later, both roles move between locations L_1_ and L_2_ losing their synchronization due to delayed movement. Finally, they leave the site of events together from location L_2_, causing the final peak in the correlation and the close proximity.

### Area coverage

**Fig. 4:**
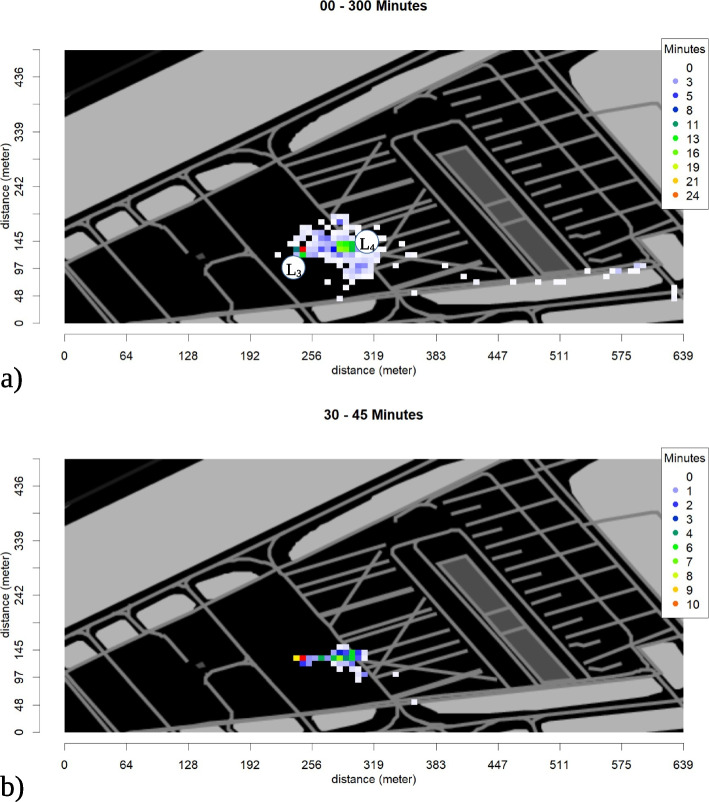
Area coverage of red SPs **a** for the whole time of simulation **b** within the time segment from 09:30 am to 09:45 am at the arrival of the senior emergency physician and the transport control officer. The *X*- and *Y*-axes show the length of the area in meters. The color marks the time SPs of the red category have been located in each square

The area coverage of the red SPs is shown in Fig. 4. The locations of the SPs for the whole simulation are shown in Fig. 4a), while Fig. 4b) shows the locations of the red SPs during the time segment from 09:30 am to 09:45 am, which matches the time of arrival of the senior emergency physician and the transport control officer in Table 2.

In Fig. 4a, the green and red dots indicate two peaks, at locations L_3_, representing the treatment area, and L_4_ the location of the SPs when the rescue teams arrived. Blue and white dots show that SPs have just passed through this area. The red dot shows that over the time of the simulation SPs of the category red were located within this square for nearly 24 min.

In Fig. 4b, the area coverage of red SPs during the arrival of the senior emergency physician is shown, giving an explanation for the movement around location L_1_, which is close to the treatment and the transport area of the SPs around that time. The observer logs in Table 2 verify the location of the senior emergency physician in the treatment area between 66 and 70 min after the start of the simulation.

Area coverage images can also be used to indicate triage errors. For example, the area coverage of the yellow SPs (not shown in the figures) shows SP no. 9 which was already verified as under-triaged in the triage logs in Table 1, as being located in a group of green SPs, and leaving the site of events together with the green group.

## Discussion

The study addressed the question of whether GPS data can be used to represent the behavior of participants during an MCI simulation. This was addressed by comparison of GPS-derived parameters with observations logged by observers. There was generally very good agreement between events observed by GPS and observer logs. The use of GPS could therefore replace some observers in MCI events, reducing costs, as well as potential observer influence on the simulation. In fact, GPS data provides information that is not available from observers, in particular, a general overview of the simulation.

In [11] the DIORAMA system was introduced, which included training for the EMS involved in the simulation. Their study showed that electronic support systems can have a positive effect on the simulation outcome and that the system was accepted by the participants.

The system introduced in this study was not intended for real-time analysis, although the methods could be extended.

Ozella et al. [17] used wearable proximity sensors to track the movement of SPs during a simulated collapse of a building, to measure the contact time between nurses, doctors, and SPs. Our observations during the MCI simulation confirm their conclusion that proximity sensors or, in our case, GPS loggers do not disturb the simulation process and preserve the immersion of the participants. In the current study, a short introduction to the GPS-loggers was given prior to each simulation but during the simulation, the devices were hidden in pockets.

### Accuracy

The high accuracy of GPS-logger data was already demonstrated in [14] and was shown again in this simulation by the comparison of observer logs and movement data.

### Triage judgment

By analyzing the times SPs left the site of events, possible misjudgments in the triage process could be identified. Observer logs and triage protocols were used to verify the results. Although the methods could not identify the reason why incorrect triage categories had been assigned to the SPs, they can draw attention to these events. As the times of exit from the scene are recorded by the technology, it would be possible to focus the attention of the observers more on the triage itself or to reduce the number of observers overall.

### Communication

The movement correlation in combination with maps of the tactical movement of participants provides an oversight of their behavior. In combination with the area coverage of the SPs, it is also possible to explain the focus of the movement on certain areas. Although the existence or quality of communication could not be assessed from the movement patterns, it could be a priority for observers in the future with movement captured using the methods presented.

### Synchronized temporal data

The temporal segmentation of the simulation based on events allows for a simple comparison between the results of the different methods and between different simulations. Summarizing the movement correlation in 15-min timespans means that short events cannot be recorded. However, this study was rather designed to investigate trends. The events used for automatic segmentation can be based on movement or observer data and can easily be modified. The events automatically created by GPS data could be verified using the observer logs.

### Added value of GPS

The evaluation of observer data showed that there were occasional gaps in the data. For example, the arrival times of the simulated patients at the simulated hospital were not recorded by the observers (although the instructions were clear in this regard). The GPS data was able to fill in the missing data.

Although the observer logs were used in this study to confirm the results of the GPS measurements, there were many cases in which the GPS measurements could be used to complete and augment logged observations. Indeed, it is likely that positional information using such tools in the future will greatly exceed that which could be logged by local observers.

### GPS limitations and other non-invasive tools

The current approach of using GPS loggers is limited to outdoor simulations, but methods to locate persons inside of buildings, using for example smartphones, exist [18]. The methods presented to analyze motion in MCI simulations are based on position coordinates that could also be provided by sources other than GPS.

### Data safety and speed restrictions

The raw data from the GPS loggers has to be manually transferred to a database and the data manually labeled. As no data is transported via a wired or wireless network, it cannot be intercepted by third parties. But the manual process takes several hours, making it impossible to use the results in an immediate post-exercise debriefing („hot debriefing “). We have developed an application for Android smartphones that is capable of collecting GPS, Wi-Fi, and Bluetooth data that allows live data collection and its transfer by e-mail. However, this application has not yet been used in a simulation exercise.

## Conclusions

This study shows that GPS tracking methods can be used to analyze the behavior of participants and assess triage decisions made during an MCI simulation. The introduction of such remote tracking technology could allow observers to focus on more specific tasks during the simulation and the number of observers could also be reduced, thereby reducing potential observer interference and bias.

Patient transport can be monitored during the simulation (even in ambulances) and can indicate the assignment of incorrect triage categories. Movement correlations between participants allow an assessment of their cooperation during a simulation.

Future research is aimed at exploring the communication structures during MCI simulations. In this way, it may be possible to find methods that allow a quality assessment of the communication via the movement patterns. Movement patterns allow the automatic recognition of higher-level communication structures.

## Data Availability

The datasets used and/or analyzed during the current study are available from the corresponding author on reasonable request.

## Abbreviations

EMS: Emergency medical service
GPS: Global positioning system
MCI: Mass casualty incident
mSTaRT: Modified Simple Triage and Rapid Treatment
RFID: Radio frequency identification
SP: Simulated patient
